# A Breathtaking Hernia: A Giant Hiatal Hernia Masquerading as Poorly Controlled Asthma

**DOI:** 10.7759/cureus.22268

**Published:** 2022-02-16

**Authors:** Abhinav Karan, Hui Jun Guo, Kintin Ng, Christopher Izzo

**Affiliations:** 1 Internal Medicine, University of Florida College of Medicine, Jacksonville, USA

**Keywords:** hiatal hernia, paraesophageal hernia, sliding hernia, pulmonary edema, gerd, heart failure, asthma, recurrent wheezing

## Abstract

A 93-year-old female presented with persistent shortness of breath and wheezing since the consumption of a meal. Her past medical history is significant for a clinical diagnosis of asthma at the age of 88 years, without pulmonary function testing, complicated by several prior visits to the emergency department (ED) for recurrent exacerbations. Multiple bronchodilators in the ED provided only minimal improvement in her symptoms. Chest imaging eventually revealed a giant, fluid-filled hiatal hernia exhibiting a compressive effect on the posterior aspect of the left atrium. The etiology of the patient's airway bronchoconstriction was likely multifactorial. We hypothesize that the extrinsic, dynamic compression of the bronchial tree by the peristaltic motion of the hiatal hernia, microaspiration from gastroesophageal reflux, and peribronchial edema from left atrial compression accounted for our patient's unique presentation. An outpatient methacholine challenge test eventually excluded bronchial asthma. Although she was considered a poor surgical candidate, she has had no further recurrences of her symptoms with counseling on conservative lifestyle changes. This case serves to highlight the heterogeneity in presentations of hiatal hernias, particularly in elderly females. Furthermore, it remains prudent to maintain a broad differential for wheezing, as evidenced by our patient who was previously managed for a number of years as poorly controlled asthma.

## Introduction

A giant hiatal hernia presents a diagnostic and therapeutic dilemma for most clinicians, often being found incidentally on chest imaging. The vast majority of hiatal hernias are asymptomatic, however, a minority of individuals can present with symptoms of gastroesophageal reflux disease [1]. Here, we present a unique case of a patient with a giant hiatal hernia who was being managed for a number of years as poorly controlled asthma, along with a brief review of the literature.

## Case presentation

A 93-year-old female presented to the emergency department with persistent shortness of breath and wheezing since consumption of a meal. Her past medical history is significant for hypertension, osteoarthritis, and a clinical diagnosis of bronchial asthma at the age of 88 years, without prior pulmonary function testing. She describes a complicated course, with multiple visits to the emergency department for recurrent episodes of dyspnea and wheezing despite bronchodilator and inhaled corticosteroid therapy. She is compliant with both a short-acting beta-agonist inhaler and an inhaled corticosteroid at home, which she uses prudently, and has received multiple courses of prednisone for presumed asthma exacerbations in her prior visits to the emergency department. She notes that she often presents to the ED for significant shortness of breath and audible wheezing, usually after a large meal, that only resolves after prolonged bronchodilator nebulization. She had not been prescribed any angiotensin-converting enzyme inhibitors, proton pump inhibitors, or H2 receptor blockers prior to presentation.

At the time of presentation, her vital signs were notable for tachycardia with a pulse of 110, and hypoxia with oxygen saturation of 86%, requiring placement of full-face bilevel positive airway pressure for titration of oxygen saturation. Her physical examination was notable for diffuse, loud wheezing auscultated throughout lung fields bilaterally, a 2/6 pansystolic murmur along the left sternal border, and mild bilateral lower limb edema. Point of care ultrasound revealed a grossly preserved ejection fraction, but some leftward bowing of the interventricular septum. Her complete blood count and comprehensive metabolic profile were unremarkable, with an N-terminal pro-B-type natriuretic peptide (NT-proBNP) of 877 pg/ml (reference range: 0-450 pg/ml).

**Figure 1:**
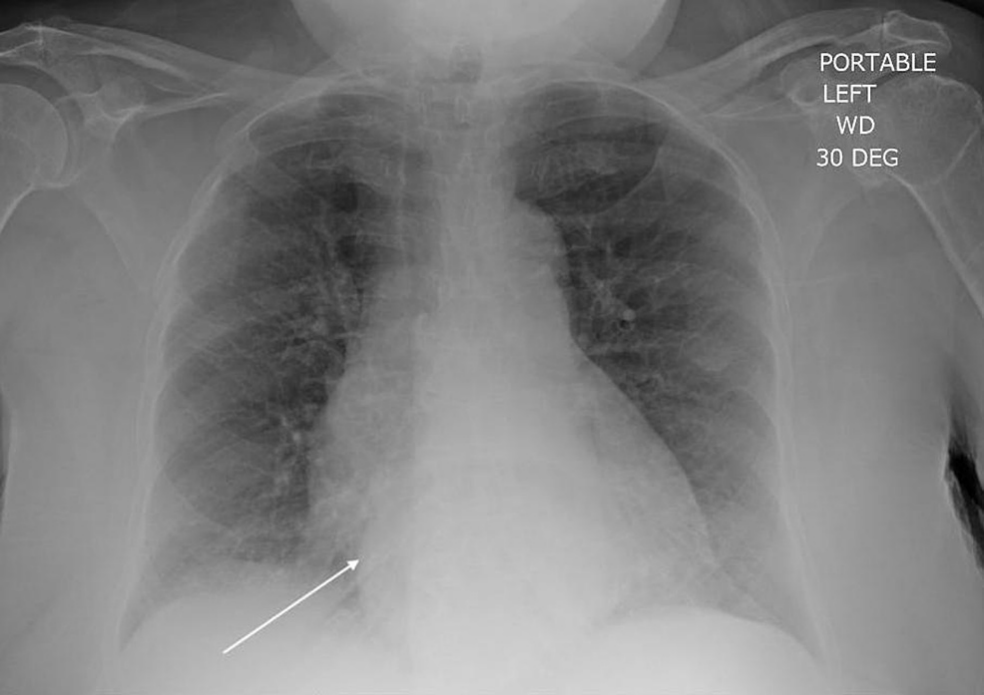
Chest x-ray showing outline of retrocardiac opacity and mild interstitial ground glass opacities bilaterally. Arrow demonstrates the extrinsic compression of the left atrium by the giant hiatal hernia.

In the emergency department, she initially received continuous nebulizers and oxygen therapy with only mild improvement in symptoms. A chest x-ray (CXR) was performed, which demonstrated mild interstitial ground glass opacities bilaterally concerning pulmonary edema, and a faint, round opacity in the retrocardiac space (Figure 1). She then received 20 mg of intravenous furosemide with a dramatic reduction in wheezing and resolution of her hypoxia, with oxygen saturations above 95% on minimal oxygen therapy. A presumptive diagnosis of decompensated heart failure was made and the patient was admitted for further management. At that time, primary asthma was low on the differential due to the atypical age of diagnosis, lack of confirmatory pulmonary function testing, poor response to bronchodilator and corticosteroid therapy, and the unusual trigger of wheezing on meal consumption. Her wheezing was initially presumed to be secondary to cardiac asthma due to her excellent response to diuretic therapy. A swallow study was done which excluded signs of aspiration.

**Figure 2:**
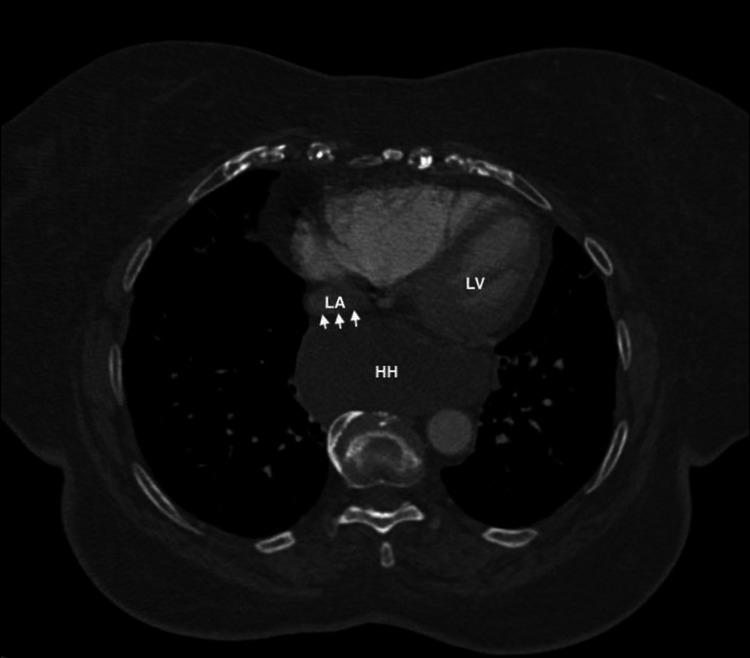
Axial view of CT chest showing compression of left atrium by a giant hiatal hernia. Arrows demonstrating the extrinsic compression of the left atrium by the giant hiatal hernia. LA: left atrium; HH: hiatal hernia; LV: left ventricle

**Figure 3:**
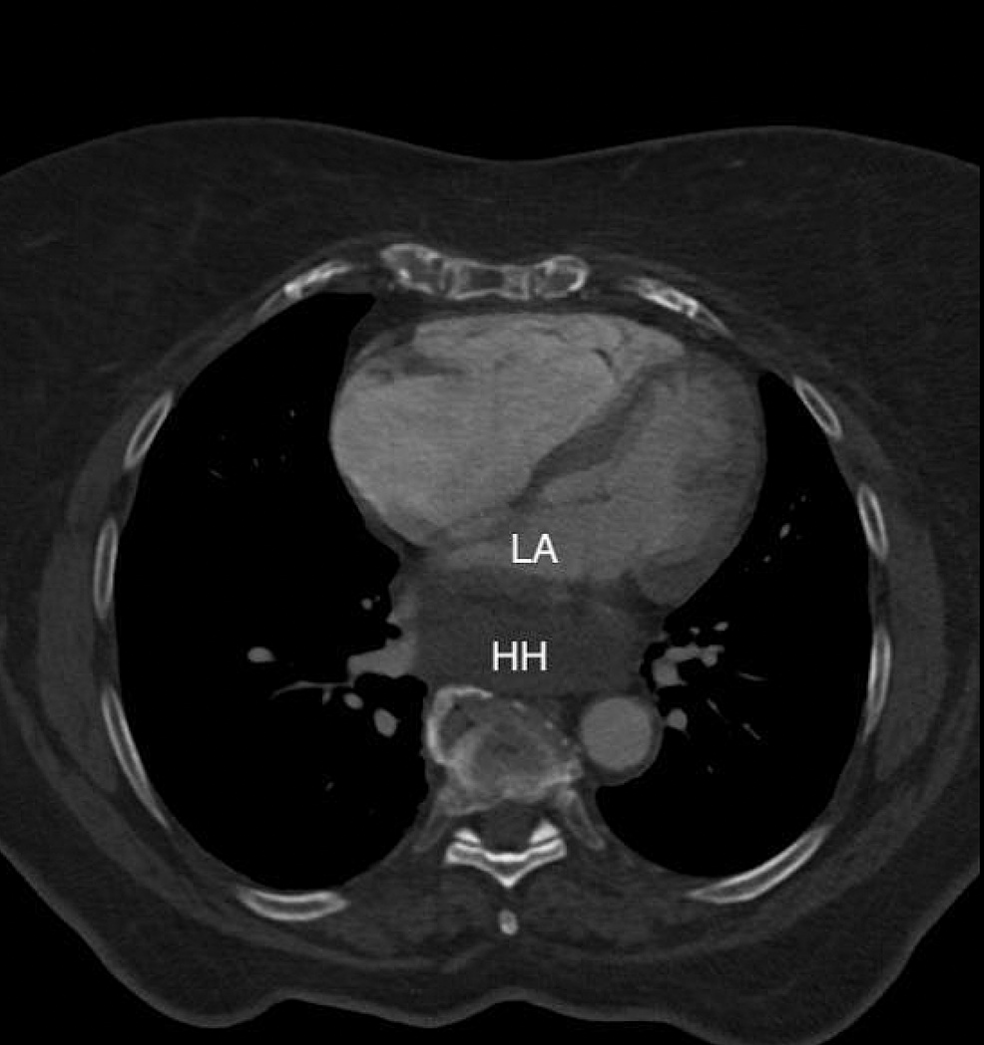
Alternate axial view of CT chest with compression of left atrium by a giant hiatal hernia. LA: left atrium; HH: hiatal hernia

**Figure 4:**
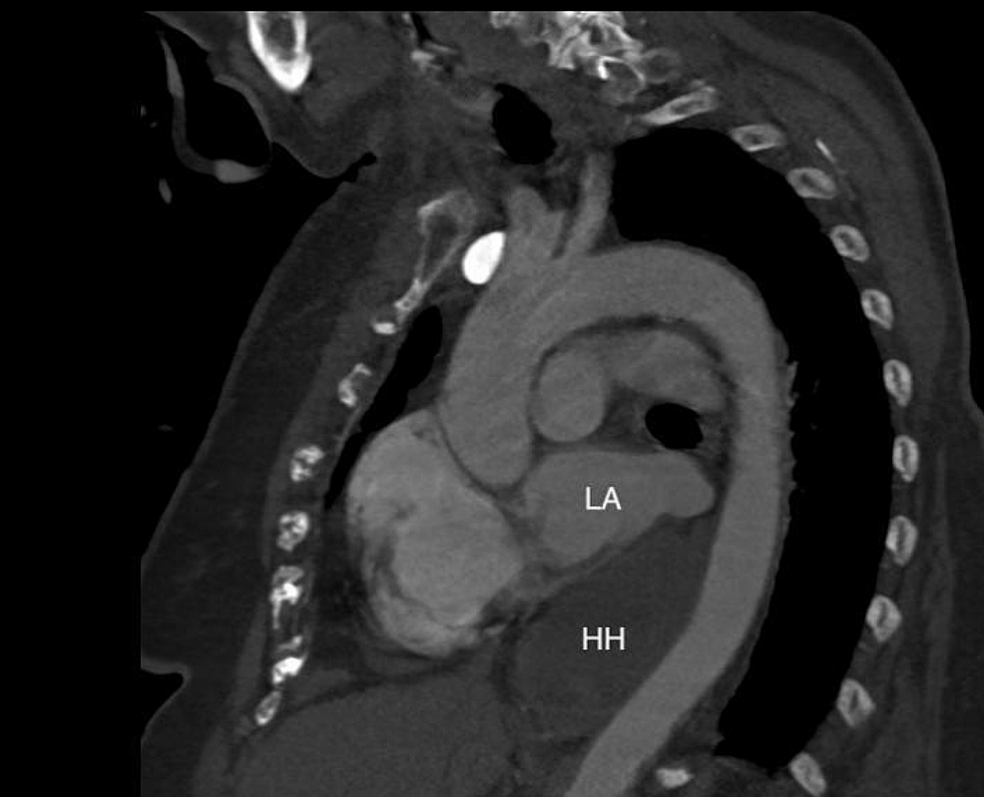
Sagittal view of CT chest with compression of the posterior left atrium by a giant hiatal hernia. LA: left atrium; HH: hiatal hernia

**Figure 5:**
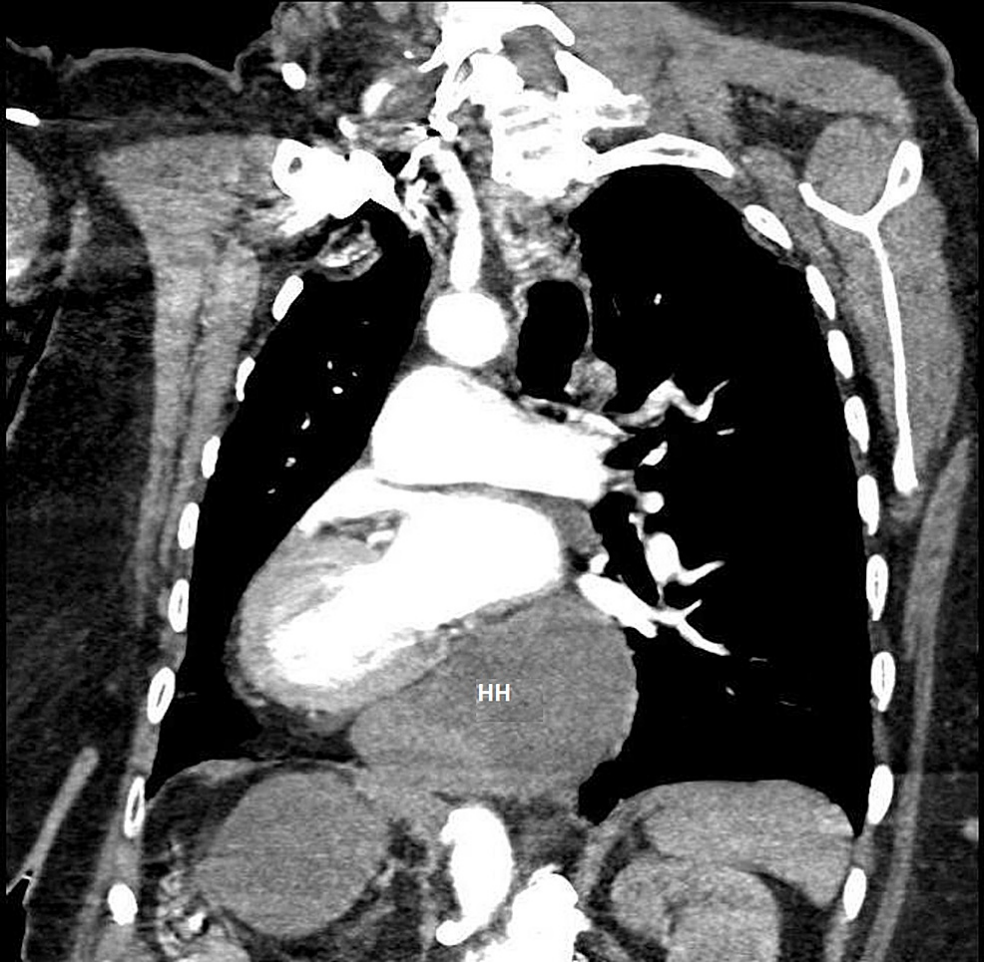
Right pulmonary artery oblique view demonstrating giant hiatal hernia with extrinsic compression of left atrium and left ventricle. HH: hiatal hernia

Due to persistent tachycardia and initial presentation of hypoxia, there was a concern for a pulmonary embolism, so a computed tomography pulmonary angiogram study (CTPA) was ordered and no emboli were found. However, it did reveal right ventricular enlargement and most notably, a distended, fluid-filled giant hiatal hernia exerting extrinsic mass effect on the posterior aspect of her heart, particularly the left atrium (Figures 2-5).

**Figure 6:**
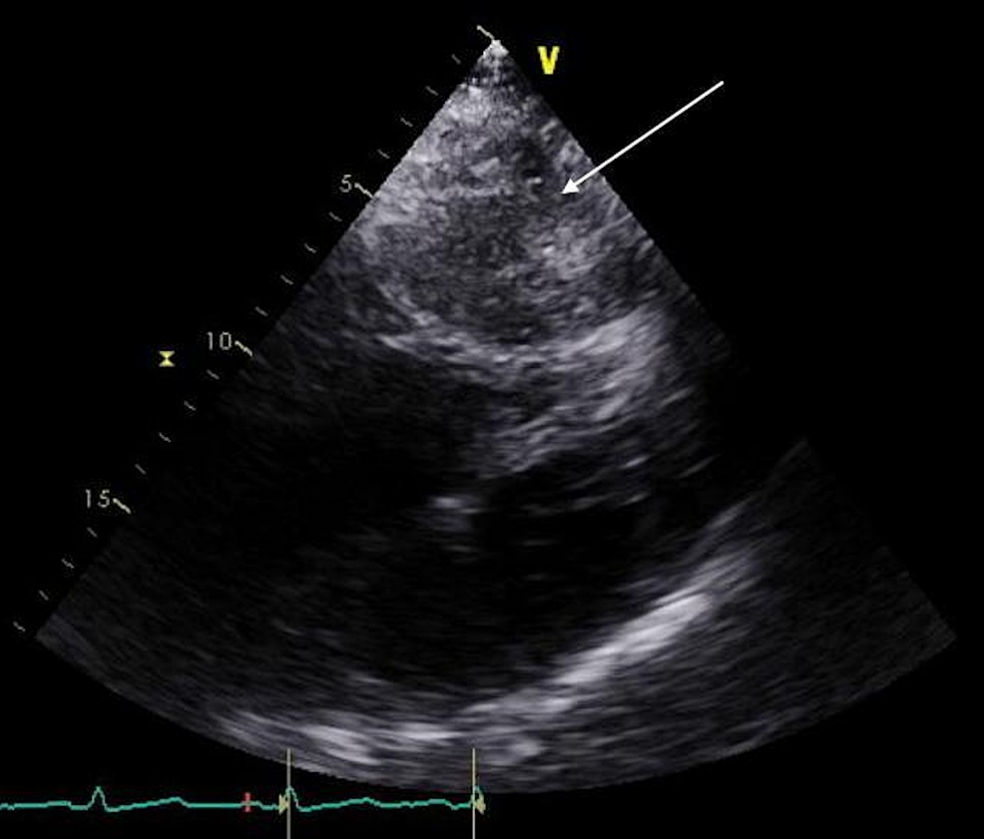
Subxiphoid view of TTE with visible hiatal hernia exhibiting compressive effect on left atrium and left ventricle (arrow). TTE: transthoracic echocardiogram

A formal transthoracic echocardiogram (TTE) revealed an ejection fraction of 60-65%, a small left ventricular and left atrial cavity, and a visible hiatal hernia in the subxiphoid view (Figure 6). An increased pulmonary artery systolic pressure of 51 mmHg, with moderate tricuspid regurgitation and an enlarged right ventricle, was also noted. She was referred for surgical intervention; however, due to her age and comorbidities, she was deemed a poor surgical candidate. Instead, she was counseled on conservative strategies to reduce the incidence of her symptoms. These measures included sleeping in an upright position after meals, smaller meal volumes, and avoidance of late-night meals. She was also started on proton pump inhibitor therapy and alginate-based antacid therapy. An outpatient methacholine challenge test was performed to exclude any element of bronchial asthma, which the patient did not have. On outpatient follow-up, she reported a significant improvement in her symptoms and has since had no further episodes of wheezing following the establishment of her lifestyle changes.

## Discussion

A uniform definition for a giant hiatal hernia (HH) does not exist yet. While they may be variously defined, there is some agreement that they refer to hernias in which >30-50% of the stomach lies within the thoracic cavity, with or without any abdominal viscera [2]. They are rare, accounting for 0.3% of all hiatal hernias, and while their presentation sounds dramatic, the majority of these cases are either asymptomatic or in those very few individuals, present with symptoms of gastrointestinal reflux disease, most commonly being found incidentally on chest imaging performed for another indication as with our case [2].

Hiatal hernias are generally divided into four different types based on their mechanism and contents within the hiatal hernias. Type I hiatal hernias are by far the most common, accounting for 85-95% of all cases, and occur when dilation of the diaphragmatic hiatus allows upwards herniation of the stomach cardia and the gastroesophageal junction [1]. Type II hiatal hernias are broadly referred to as paraesophageal hernias, and occur when there is an associated defect in the phrenoesophageal membrane. This results in the gastroesophageal junction remaining fixed in position due to its attachment to the preaortic fascia, but the stomach herniating adjacent to the esophagus. Type III hernias broadly have a mixed mechanism and involve components of type I and type II hernias, and type IV hernias refer to any hiatal hernia with the presence of contents other than the stomach, such as the colon, omentum, or small bowel, within the hernia sac. More than 90% of all giant hiatal hernias are type III hiatal hernias, with herniation of extra-gastric contents not being uncommon [1].

Our patient presented with the primary symptom of wheezing after consumption of meals, initially managed as asthma. The underlying mechanism of this presentation warrants further discussion. In our case, it is likely that the extrinsic compression of the left atrium by the distended hiatal hernia resulted in a mechanical elevation in the pulmonary and right-sided cardiac pressures leading to both her mild pulmonary and peripheral edema. The etiology of her airway bronchoconstriction is likely multifactorial. We hypothesized that the extrinsic, dynamic compression of her bronchial tree from the peristaltic motion of the distended hiatal hernia, particularly on the consumption of meals, likely caused intermittent airway obstruction mimicking that of asthma exacerbation. In addition, there is likely a component of microaspiration from gastroesophageal reflux not detected on her swallowing studies, and peribronchial edema from the significant extrinsic left atrial compression by her giant hiatal hernia.

On review of the current literature on patients with a giant hiatal hernia presenting with primary respiratory complaints, without gastrointestinal symptoms, only a handful of case reports are noted, and none with the primary symptom of recurrent episodes of wheezing. These are summarized in Table 1 below [3-7]. While a small cohort, some interesting observations about the trends in presentations can be observed. As with our patient, the majority of patients presenting with atypical symptoms of a hiatal hernia are elderly, with the majority above the age of 80 years and notable female predominance. The reasoning behind this apparent trend is unknown and certainly represents a point of further research on hiatal hernias. Furthermore, while clinicians should be aware of this heterogeneity in presentations, epidemiological studies are necessary to determine whether this observed trend is statistically significant. The impact of a giant hiatal hernia on pulmonary function testing is recognized, with some literature demonstrating that surgical repair of a giant hiatal hernia can result in the improvement of a patient’s forced expiratory volume in 1 second (FEV1) by >20%, with a positive correlation between the size of the giant hiatal hernia, and degree of improvement in FEV1 [8]. There is also improvement in FVC and TLC, suggesting that surgical management of symptomatic giant hiatal hernias is an effective modality for therapy [8].

**Table 1:** Characteristics of patients described in prior case reports of patients with giant hiatal hernias presenting with respiratory complaints without gastrointestinal symptoms.

Author	Mirdamadi and Arasteh [3]	Torres et al. [4]	Chou and Su [5]	Sahin et al. [6]	Wongrakpanich et al. [7]
Year	2010	2013	2014	2015	2016
Age (years)	78	82	86	84	88
Gender	Female	Female	Female	Female	Female
Presenting symptoms	Paroxysmal nocturnal dyspnea	Exertional dyspnea	Exertional dyspnea	Progressive dyspnea	Progressive dyspnea
Chest x-ray	Large epicardial fat pad	N/A	Cardiomegaly with mediastinal widening	Mediastinal widening with retrocardiac mass and air-fluid level	Opacity in left lower lobe
CT chest	Large hiatal hernia	Hiatal herniation without any structural lung disease	Hiatal hernia	Large hiatal hernia with mild compression of the left atrium	Giant hiatal hernia containing stomach, pancreas, duodenum
Management and outcome	N/A	Patient refused surgical management	Patient refused surgical management	Patient refused surgical management	Patient refused surgical management

For patients who refused surgery, or are poor surgical candidates due to age and existing comorbidities, there are still benefits to conservative management. These include a reduction in meal volumes, remaining upright after meals, and the addition of a proton pump inhibitor. These strategies are typically effective for the majority of patients with a type I hiatal hernia, where surgery is not typically warranted. In our case, it was theorized that small volume meals will aid in the reduction of the degree of peristaltic dilation of the hiatal hernia that occurs with meal consumption, the primary trigger for her episodes of wheezing. Proton pump inhibitor therapy will be effective in reducing any events of microaspiration from underlying gastroesophageal reflux from the underlying hiatal hernia. Additionally, arginate-based-suspensions as an adjunct to proton pump inhibitor therapy is frequently performed and has been shown to decrease acid reflux events within the first hour of administration [9]. This is presumed beneficial for our patient whose major trigger for wheezing was meal consumption. 

While a giant hiatal hernia has previously been reported very infrequently to present atypically with acute heart failure [10], arrhythmias, angina pectoris, or symptoms of exercise impairment [7], this case highlights an additional important atypical presentation of giant hiatal hernias. While shortness of breath and wheezing typically portends a consideration of bronchial asthma, it is important to consider that they may be representative of an underlying hiatal hernia, particularly in elderly patients with new-onset wheezing. 

## Conclusions

A giant hiatal hernia is a rarely encountered entity with an apparent trend towards atypical presentations in elderly female patients. We highlight a unique presentation of a patient with recurrent episodes of wheezing, previously being treated for a number of years as poorly controlled asthma, later discovered to have a giant hiatal hernia. With lifestyle changes and conservative management, the patient has had no further recurrences of her symptoms. This case serves to highlight the heterogeneity of presentations of giant hiatal hernias, particularly for elderly patients, representing a diagnostic and therapeutic conundrum for this patient population. Furthermore, although pulmonary causes are frequently the attributed cause of wheezing, clinicians must be prudent to include both cardiac and gastrointestinal causes in the differential, particularly for elderly patients.
